# Supplementary material for: Genetic and Epigenetic Profiles of Polycystic Ovarian Syndrome and In Vitro Bisphenol Exposure in a Human Granulosa Cell Model
Source: Biomedicines. 2024 Jan 21;12(1):237. doi: 10.3390/biomedicines12010237 (PMC10813104; doi:10.3390/biomedicines12010237)
Supplement: Supplementary file 1 [file biomedicines-12-00237-s001.zip › biomedicines-2812366-supplementary.pdf]

<u>Supplementary Materials</u>

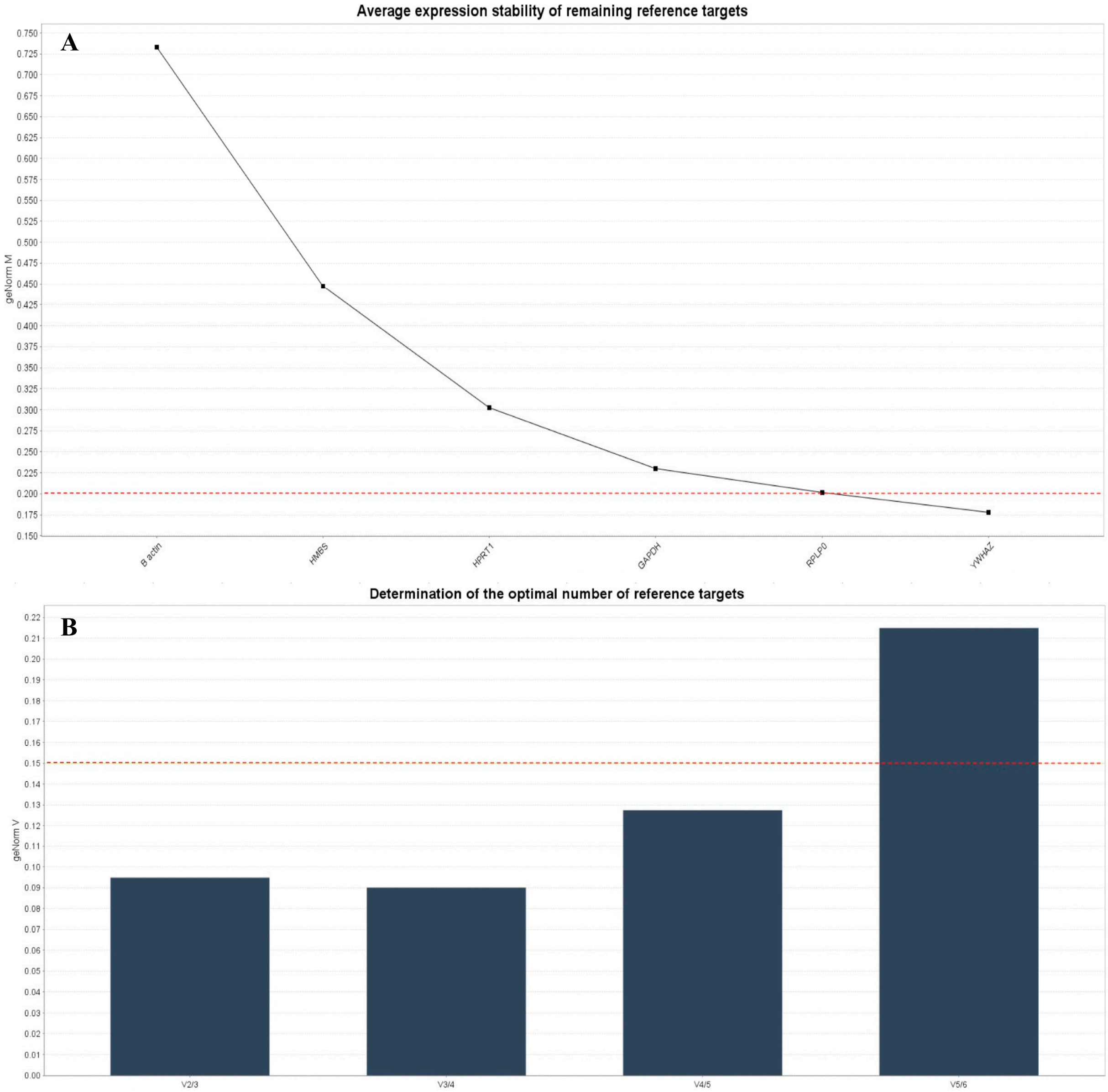

Figure S1: Stability of Reference Targets. **(A)** High reference target stability (geNorm M $\leq$ 0.2) represented for reference targets RPLP0 and YWHAZ, below the dotted red line. **(B)** Suggested Number of Reference Targets. The optimal number of reference targets forGeNorm analysis was determined to be 2 (geNorm < 0.15).
